# Supplementary material for: Clinical and Histopathologic Features of a Feline SARS-CoV-2 Infection Model Are Analogous to Acute COVID-19 in Humans
Source: Viruses. 2021 Aug 5;13(8):1550. doi: 10.3390/v13081550 (PMC8402899; doi:10.3390/v13081550)
Supplement: Supplementary file 1 [file viruses-13-01550-s001.zip › viruses-1315183-supplementary CM edits.pdf]

viruses

Supplementary Materials

Clinical and Histopathologic Features of a Feline SARS-CoV-2 Infection Model Are Analogous to Acute COVID-19 in Humans

Jennifer M. Rudd [1], Miruthula Tamil Selvan [1], Shannon Cowan [1], Yun-Fan Kao [1], Cecily C. Midkiff [2], Sai Narayanan [3], Akhilesh Ramachandran [3], Jerry W. Ritchey [1] and Craig A. Miller [1,*]

[1] Department of Veterinary Pathobiology, College of Veterinary Medicine, Oklahoma State University, Stillwater, OK 74078, USA; jennifer.rudd@okstate.edu (J.M.R.); mtamils@ostatemail.okstate.edu (M.T.S.); shannon.r.cowan@okstate.edu (S.C.); vertoiseaux@gmail.com (Y.-F.K.); jerry.ritchey@okstate.edu (J.W.R.)
[2] Division of Comparative Pathology, Tulane National Primate Research Center, Tulane University, Covington, LA 70433, USA; cconerly@tulane.edu
[3] Oklahoma Animal Disease Diagnostic Laboratory, College of Veterinary Medicine, Oklahoma State University, Stillwater, OK 74078, USA; rakhile@okstate.edu (A.R.); ssankar@okstate.edu (S.N.)
* Correspondence: craig.miller@okstate.edu

Citation: Rudd, J.M.; Tamil Selvan, M.; Cowan, S.; Kao, Y-F.; Midkiff, C.C.; Ramachandran, A.; Narayanan, S.; Ritchey, J.W.; Miller, C.A. Clinical and Histopathologic Features of a Feline SARS-CoV-2 Infection Model Are Analogous to Acute COVID-19 in Humans. *Viruses* **2021**, *13*, 1550. https://doi.org/10.3390/v13081550

Academic Editor(s): Francisco Javier Salguero

Received: 10 July 2021
Accepted: 2 August 2021
Published: 10 August 2021

Publisher's Note: MDPI stays neutral with regard to jurisdictional claims in published maps and institutional affiliations.

Copyright: © 2021 by the authors. Licensee MDPI, Basel, Switzerland. This article is an open access article distributed under the terms and conditions of the Creative Commons Attribution (CC BY) license (http://creativecommons.org/licenses/by/4.0/).

Table S1. Clinical Scoring Data Summary. (**A**) Total number of cats from each group exhibiting clinical signs during the 8-day study. (**B**) Total clinical scores recorded each day per individual cat. Statistical analysis and raw scoring data for each day is included in Supporting Information.

A.

Total # Cats with Specific Clinical Sign During Study		
**Clinical Sign**	**PBS(*n* = 6)**	**SARS-CoV-2 (*n* = 12)**
Weight loss	1	5
Reduced activity	0	12
Altered behavior	0	7
Hyperthermia	0	8
Tachypnea or Dyspnea	0	12
Ocular or Nasal Discharge	0	0
Coughing/Wheezing	0	4

B.

	Total Clinical Score (Sum of Parameter Scores)									
**Animal ID**	**Day 0**	**Day 1**	**Day 2**	**Day 3**	**Day 4**	**Day 5**	**Day 6**	**Day 7**	**Day 8**	**Peak Clinical Score**
Control 1 (0109)	0	0	0	0	0	-	-	-	-	**0**
Control 2 (0575)	0	0	0	0	0	-	-	-	-	**0**
Control 3 (0664)	0	0	0	0	0	-	-	-	-	**0**
Control 4 (1024)	0	0	0	0	0	0	0	0	0	**0**
Control 5 (0923)	0	0	0	0	0	0	0	0	0	**0**
Control 6 (0907)	0	0	0	0	0	1	0	0	0	**0**
Infected 1 (9491)	0	0	0	0	2	-	-	-	-	**2**
Infected 2 (0613)	0	3	3	2	2	-	-	-	-	**3**
Infected 3 (9687)	0	0	0	0	2	-	-	-	-	**2**
Infected 4 (9857)	0	0	0	0	2	-	-	-	-	**2**
Infected 5 (0452)	0	0	2	7	7	-	-	-	-	**7**
Infected 6 (1474)	0	1	5	3	6	-	-	-	-	**6**
Infected 7 (1440)	0	1	0	0	6	4	2	2	3	**6**
Infected 8 (0800)	0	2	0	0	2	4	2	3	2	**4**
Infected 9 (0559)	0	2	2	1	9	4	3	6	6	**9**
Infected 10 (1130)	0	0	0	3	2	5	2	2	2	**5**
Infected 11 (0095)	0	0	1	0	2	1	2	2	3	**3**
Infected 12 (0842)	0	1	1	0	2	2	2	2	2	**2**

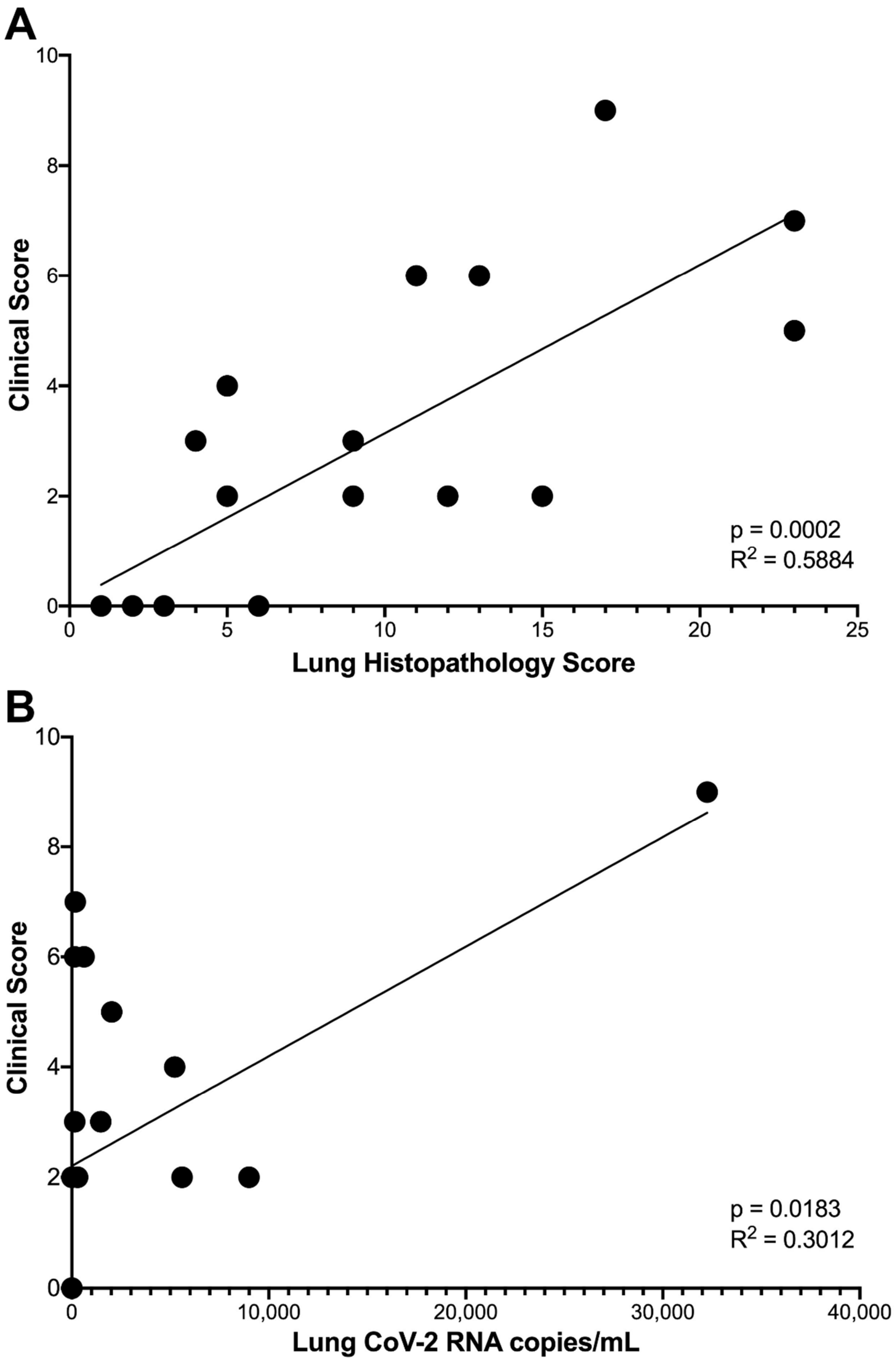

Figure S1. Positive linear correlation between histopathologic score and peak clinical score. (**A**) A positive linear correlation exists between more severe histologic scores from analyzed lung tissue and peak clinical score identified in infected cats. Cats with more severe clinical disease were also more likely to have more severe pulmonary pathology at the time of necropsy. $p = 0.0002$; $R^2 = 0.5884$. (**B**) This graph details the correlation between peak clinical score and lung SARS-CoV-2 RNA viral load. This correlation was significant ($p = 0.0183$), but the measure of fit was below 50% for variance ($R^2 = 0.3012$). Data was analyzed via Pearson Correlation (Table S2).

Table S2. Lung tissue histopathology scores based on pathologic features reported in human COVID-19 patients [1–4]. All tissues were assigned a quantitative histologic score of 0–4 based on previously documented criteria [5,6]. Lung tissues were scored as described in methods by a board-certified veterinary anatomic pathologist blinded to study groups. Histopathologic scores were analyzed using two-way ANOVA and Tukey's multiple comparison test.

	Cat ID	Alveolar Damage	Alveolar Fibrin	Serous Exudate/ Pulmonary Edema	Perivascular Infiltrates	Alveolar Histiocytosis	Type II Pneumocyte Hyperplasia	Syncytia	Thrombosis	Vasculitis	Total Histopathology Score
Uninfected	0109	0	0	1	1	1	0	0	0	0	**3**
	0575	0	0	0	0	1	0	0	0	0	**1**
	0664	0	0	1	1	0	0	0	0	0	**2**
	0907	0	0	1	0	1	0	0	0	0	**2**
	0923	0	0	1	2	2	1	0	0	0	**6**
	1024	0	0	0	1	1	0	0	0	0	**2**
Day 4 p.i.	0452	3	3	4	4	4	3	2	0	0	**23**
	0613	0	0	2	2	0	0	0	0	0	**4**
	1474	2	0	0	3	4	2	2	0	0	**13**
	9491	2	0	4	3	3	0	0	0	0	**12**
	9687	2	0	1	3	3	0	0	0	0	**9**
	9857	3	2	3	3	4	0	0	0	0	**15**
Day 8 p.i.	0095	1	0	2	3	3	0	0	0	0	**9**
	0559	2	2	4	3	4	0	0	2	1	**17**
	0800	0	0	1	1	1	0	0	2	0	**5**
	0842	0	0	2	1	2	0	0	0	0	**5**
	1130	3	2	4	4	4	3	3	0	2	**23**
	1440	1	1	3	3	3	0	0	0	0	**11**

p.i. = post infection.

Table S3. Total SARS-CoV-2 viral RNA and fACE2 RNA (copy number/mL) detected in each tissue type. ANOVA was used to compare differences in SARS-CoV-2 viral load and ACE2 RNA among uninfected and SARS-CoV-2-infected individuals, between sample type, for each tissue individually, and between tissues. RNA copy number per mL and statistical analysis are reported in the above table. TBLN = tracheobronchial lymph node. Vertical text in 1st column indicates group.

		CoV-2 Viral RNA Copies/mL					ACE2 RNA Copies/mL				
	Cat ID	Nasal Turbinates	TB LN	Distal Trachea	Kidney	Lung	Nasal Turbinates	TB LN	Distal Trachea	Kidney	Lung
Uninfected	0109	0	0	0	0	0	4178	0	1511	42,667	133
	0575	0	0	0	0	0	138,889	0	133	32,667	778
	0664	0	0	0	0	0	3956	0	1711	189,333	0
	0907	0	0	0	0	0	3200	311	0	192,000	578
	0923	0	0	0	0	0	1578	1111	533	147,778	0
	1024	0	0	0	0	0	4489	0	133	51,778	0
Day 4 p.i.	0452	2755	214,666	7333	267	488,667	778	178	0	242,444	0
	0613	378	244	14,444	3178	3778	667	0	133	263,111	133
	1474	1622	524,444	178	1400	594,889	1289	0	467	189,556	533
	9491	104,667	1889	0	622	1489	733	0	133	282,000	133
	9687	16,000	253,555	2445	1644	866	64,889	0	400	833,333	0
	9857	47,111	8889	4334	489	503,778	25,333	267	0	206,667	0
Day 8 p.i.	0095	0	226,666	32,000	1755	1133	2644	422	133	148,222	0
	0559	178	384,445	200	222	28,666	2356	0	0	150,667	0
	0800	712	22,667	63,778	1711	8000	8222	10,444	267	129,333	667
	0842	956	189,556	0	2889	778	156	2600	133	65,556	0
	1130	11,111	334,000	600	1889	9267	1000	0	267	76,222	133
	1440	3755	768,889	24,000	644	6444	1822	133	0	58,444	0

* TB LN = tracheobronchial lymph node, p.i. = post infection

Table S4. Relationship between feline ACE2 expression and tissue type (Kruskal-Wallis test). (**A**) Sham-inoculated control animals; (**B**) SARS-CoV-2-infected animals, 4 days post infection (dpi); (**C**) SARS-CoV-2-infected animals, 8 dpi. * $p < 0.05$; ** $p < 0.01$; *** $p < 0.001$; **** $p < 0.0001$, ns = not-significant

A. Uninfected Control Animals (n = 6): ACE2 RNA Expression by Tissue Type

Kruskal-Wallis Test	Mean rank diff.	Significant?	Summary	P value
Nasal Turbinates vs. TbLn	14.17	Yes	**	0.0049
Nasal Turbinates vs. Distal Trachea	9.583	No	ns	0.0569
Nasal Turbinates vs. Kidney	−5.167	No	ns	0.3046
Nasal Turbinates vs. Lung	13.08	Yes	**	0.0093
TbLn vs. Distal Trachea	−4.583	No	ns	0.3624
TbLn vs. Kidney	−19.33	Yes	***	0.0001
TbLn vs. Lung	−1.083	No	ns	0.8296
Distal Trachea vs. Kidney	−14.75	Yes	**	0.0034
Distal Trachea vs. Lung	3.5	No	ns	0.4868
Kidney vs. Lung	18.25	Yes	***	0.0003

Test details	Mean rank 1	Mean rank 2	Mean rank diff.	n1	n2	Z
Nasal Turbinates vs. TbLn	21.83	7.667	14.17	6	6	2.815
Nasal Turbinates vs. Distal Trachea	21.83	12.25	9.583	6	6	1.904
Nasal Turbinates vs. Kidney	21.83	27	−5.167	6	6	1.027
Nasal Turbinates vs. Lung	21.83	8.75	13.08	6	6	2.6
TbLn vs. Distal Trachea	7.667	12.25	−4.583	6	6	0.9107
TbLn vs. Kidney	7.667	27	−19.33	6	6	3.842
TbLn vs. Lung	7.667	8.75	−1.083	6	6	0.2153
Distal Trachea vs. Kidney	12.25	27	−14.75	6	6	2.931
Distal Trachea vs. Lung	12.25	8.75	3.5	6	6	0.6955
Kidney vs. Lung	27	8.75	18.25	6	6	3.626

B. SARS-CoV-2-Infected Animals, Day 4 p.i. (n = 6): ACE2 RNA Expression by Tissue Type

Kruskal-Wallis Test	Mean rank diff.	Significant?	Summary	P value
Nasal Turbinates vs. TbLn	13.33	Yes	**	0.0078
Nasal Turbinates vs. Distal Trachea	10.5	Yes	*	0.036
Nasal Turbinates vs. Kidney	−6	No	ns	0.2309
Nasal Turbinates vs. Lung	12.17	Yes	*	0.0151
TbLn vs. Distal Trachea	−2.833	No	ns	0.5716
TbLn vs. Kidney	−19.33	Yes	***	0.0001
TbLn vs. Lung	−1.167	No	ns	0.8158
Distal Trachea vs. Kidney	−16.5	Yes	***	0.001
Distal Trachea vs. Lung	1.667	No	ns	0.7393
Kidney vs. Lung	18.17	Yes	***	0.0003

Test details	Mean rank 1	Mean rank 2	Mean rank diff.	n1	n2	Z
Nasal Turbinates vs. TbLn	21.5	8.167	13.33	6	6	2.662
Nasal Turbinates vs. Distal Trachea	21.5	11	10.5	6	6	2.096
Nasal Turbinates vs. Kidney	21.5	27.5	−6	6	6	1.198
Nasal Turbinates vs. Lung	21.5	9.333	12.17	6	6	2.429
TbLn vs. Distal Trachea	8.167	11	−2.833	6	6	0.5657
TbLn vs. Kidney	8.167	27.5	−19.33	6	6	3.86
TbLn vs. Lung	8.167	9.333	−1.167	6	6	0.2329
Distal Trachea vs. Kidney	11	27.5	−16.5	6	6	3.294
Distal Trachea vs. Lung	11	9.333	1.667	6	6	0.3328
Kidney vs. Lung	27.5	9.333	18.17	6	6	3.627

C. SARS-CoV-2-Infected Animals, Day 8 p.i. (n = 6): ACE2 RNA Expression by Tissue Type

Kruskal-Wallis Test	Mean rank diff.	Significant?	Summary	P value
Nasal Turbinates vs. TbLn	5.75	No	ns	0.2529
Nasal Turbinates vs. Distal Trachea	9.333	No	ns	0.0634
Nasal Turbinates vs. Kidney	−8.333	No	ns	0.0975
Nasal Turbinates vs. Lung	11.58	Yes	*	0.0213
TbLn vs. Distal Trachea	3.583	No	ns	0.4761
TbLn vs. Kidney	−14.08	Yes	**	0.0051
TbLn vs. Lung	5.833	No	ns	0.246

Distal Trachea vs. Kidney	−17.67	Yes	***	0.0004		
Distal Trachea vs. Lung	2.25	No	ns	0.6546		
Kidney vs. Lung	19.92	Yes	****	<0.0001		
**Test details**	**Mean rank 1**	**Mean rank 2**	**Mean rank diff.**	**n1**	**n2**	**Z**
Nasal Turbinates vs. TbLn	19.17	13.42	5.75	6	6	1.143
Nasal Turbinates vs. Distal Trachea	19.17	9.833	9.333	6	6	1.856
Nasal Turbinates vs. Kidney	19.17	27.5	−8.333	6	6	1.657
Nasal Turbinates vs. Lung	19.17	7.583	11.58	6	6	2.303
TbLn vs. Distal Trachea	13.42	9.833	3.583	6	6	0.7126
TbLn vs. Kidney	13.42	27.5	−14.08	6	6	2.801
TbLn vs. Lung	13.42	7.583	5.833	6	6	1.16
Distal Trachea vs. Kidney	9.833	27.5	−17.67	6	6	3.513
Distal Trachea vs. Lung	9.833	7.583	2.25	6	6	0.4474
Kidney vs. Lung	27.5	7.583	19.92	6	6	3.961

TbLn = tracheobronchial lymph node.

References

1. Ritchey, J.W.; Levy, J.K.; Bliss, S.K.; Tompkins, W.A.; Tompkins, M.B. Constitutive expression of types 1 and 2 cytokines by alveolar macrophages from feline immunodeficiency virus-infected cats. *Vet. Immunol. Immunopathol.* **2001**, *79*, 83–100, doi:10.1016/s0165-2427(01)00250-1.
2. Polak, S.B.; Van Gool, I.C.; Cohen, D.; Jan, H.; van Paassen, J. A systematic review of pathological findings in COVID-19: A pathophysiological timeline and possible mechanisms of disease progression. *Mod. Pathol.* **2020**, *33*, 2128–2138.
3. Tian, S.; Xiong, Y.; Liu, H.; Niu, L.; Guo, J.; Liao, M.; Xiao, S.-Y. Pathological study of the 2019 novel coronavirus disease (COVID-19) through postmortem core biopsies. *Mod. Pathol.* **2020**, *33*, 1007–1014.
4. von der Thüsen, J.; van der Eerden, M. Histopathology and genetic susceptibility in COVID-19 pneumonia. *Eur. J. Clin. Invest.* **2020**, *50*, e13259.
5. Xu, Z.; Shi, L.; Wang, Y.; Zhang, J.; Huang, L.; Zhang, C.; Liu, S.; Zhao, P.; Liu, H.; Zhu, L. et al. Pathological findings of COVID-19 associated with acute respiratory distress syndrome. *Lancet Respir. Med.* **2020**, *8*, 420–422.
6. Miller, C.; Bielefeldt-Ohmann, H.; MacMillan, M.; Huitron-Resendiz, S.; Henriksen, S.; Elder, J.; VandeWoude, S. Strain-specific viral distribution and neuropathology of feline immunodeficiency virus. *Vet. Immunol. Immunop.* **2011**, *143*, 282–291.
